# The whole transcriptome regulation as a function of mitochondrial polymorphisms and aging in *Caenorhabditis elegans*

**DOI:** 10.18632/aging.102754

**Published:** 2020-02-04

**Authors:** Yuanjian Song, Yuechen Wang, Ying Li, Liang Wang, WenDa Zhang, Jing Cheng, Yao Zhu, Haoyu Zhang, Qiang Zhang, Haichen Niu, Yingwei zheng, Mengyu Liang, Mengqiong Deng, Hao Shi, Hao Wang, Fang Zhang, Zuobin Zhu

**Affiliations:** 1 Department of Genetics, Xuzhou Medical University, Xuzhou, China; 2 Medical Technology School of Xuzhou Medical University, Xuzhou, China; 3 Department of Bioinformatics, School of Medical Informatics and Engineering, Xuzhou Medical University, Xuzhou, China; 4 Department of Urology, Xuzhou Central Hospital, Xuzhou, China; 5 School of Marine Sciences, Nanjing University of Information Science and Technology, Nanjing, China; 6 Department of Biochemistry, Xuzhou Medical University, Xuzhou, China; 7 Clinical College of Xuzhou Medical University, Xuzhou, China; 8 Research Facility Center for Morphology, Xuzhou Medical University, Xuzhou, China

**Keywords:** mitochondria, aging, transcriptome, metabolome, *C. elegans*

## Abstract

Recently, mitochondrial-nuclear interaction in aging has been widely studied. However, the nuclear genome controlled by natural mitochondrial variations that influence aging has not been comprehensively understood so far. We hypothesized that mitochondrial polymorphisms could play critical roles in the aging process, probably by regulation of the whole-transcriptome expression. Our results showed that mitochondria polymorphisms not only decreased the mitochondrial mass but also miRNA, lncRNA, mRNA, circRNA and metabolite profiles. Furthermore, most genes that are associated with mitochondria show age-related expression features (P = 3.58E-35). We also constructed a differentially expressed circRNA-lncRNA-miRNA-mRNA regulatory network and a ceRNA network affected by the mitochondrial variations. In addition, Kyoto Encyclopedia of Genes and Genomes pathway analyses showed that the genes affected by the mitochondrial variation were enriched in metabolic activity. We finally constructed a multi-level regulatory network with aging which affected by the mitochondrial variation in *Caenorhabditis elegans*. The interactions between these genes and metabolites have great values for further aging research. In sum, our findings provide new evidence for understanding the molecular mechanisms of how mitochondria influence aging.

## INTRODUCTION

Mitochondria functions as an essential energy provider for eukaryotic cells and could occupy up to 20% cell volume, which makes it vital in cell growth and aging. Although mounting evidence suggests that the mitochondrial dysfunction is a central event of aging processes [1], some studies showed that mitochondrial DNA polymorphisms are associated with human longevity [2, 3]. However, the mechanism behind the association remains unclear. Recently, nuclear genes have been reported to be capable of altering mitochondrial functions with severe biological dysfunction [4], and the mtDNA genotype vary among different nuclear allelic backgrounds could influence healthy ageing [5]. Most studies of regulatory mechanism of the mitochondrial function have focused on nuclear genes. However, a recent study showed that the mitochondrial-encoded peptide could regulate nuclear gene expression in response to metabolic stress [6]. Currently, mtDNA variants can significant increase or decrease the expressivity of nuclear genes causing diseases which leads to the postulation that mitochondrial-to-nuclear signaling might accelerate the process of aging [7]. In contrast, comprehensive understanding of how mitochondria regulate the nuclear genome and then influence aging is still lacking.

The longevity of *C. elegans* is an important feature of aging and is convenient for genetic analysis, as different populations of a species can live in a standardized and uniform environment. It is now known that natural mitochondrial variant in COX1 subunit of mitochondrial complex IV (CIV) and mitochondrial physiological functions show apparent differences in between two wild isolates of the Bristol strain (N2) and the Hawaii strain (CB4856) [8]. In addition, strain CB4856 has a comparatively shorter lifespan than strain N2 [8, 9]. Dingley *et al.* speculated that mtDNA and mitochondrial-nuclear genome (mit-n) mismatch may lead to a reduction in lifespan [8]. Our previous studies also confirmed that the degree of mit-n mismatch was linked with the mitochondrial function, leading to the quantitative trait of *C. elegans* lifespan [9]. So far, it is not clear whether the shorter lifespan of CB4856 is independently caused by mitochondrial dysfunctions or the nuclear gene expressions since the nuclear genomes between CB4856 and N2 are significantly different [10]. Comprehension of how naturally occurred variations in mitochondrial DNA causing quantitative variations in phenotypic traits is a major challenge of contemporary biology. To gain further insight into mitochondrial polymorphisms is the starting point in understanding aging process. In this study, we used the wild N2 strain and the trans-nuclear cybrid *C. elegans* CN30 to examine how the natural variations of COX1 in mitochondria interacts with the nuclear genes and decreases the longevity of *C. elegans*. The nuclear transfer strain CN30 was constructed by first crossing N2 males with CB4856 hermaphrodite, then by repeatedly crossing the offspring with N2 males up to 20 generations, and finally by inbreeding up to 30 generations to generate the final CN30 strain. The strains were verified by 20 SNPs that were evenly distributed on the six pairs of chromosomes and the mitochondrial genome. Since the N2 and CN30 worms have almost the same nuclear background and they were experimented with the standardized and uniform environment, the trans-nuclear cybrid strain CN30 is a good model to study mitochondrial function and its interaction with nuclear genome.

Complex and highly ordered trait should be subject to influenced by the complex regulatory networks which could be changed by the variations [11]. Accordingly, the aging and longevity traits that normally display quantitative genetic variations should be regulated by a highly ordered biological system. Thus, we postulated that all random variations with deleterious impacts such as random noise and entropy increment, *etc.* should be under negative selection and lead to increased aging. Natural variations in mitochondria not only generate the noises in the ordered molecular regulatory network but also supply the necessary energy for the effective operation of the biological system. In this study, we proposed a hypothesis that longevity or aging as a complex trait is likely to be controlled by nuclear gene expression network, which could be further regulated by natural variations in mitochondrial DNA. Thereafter, construction of gene expression regulation networks regulated by mitochondrial variations can facilitate the understanding of the regulatory mechanism on aging.

mRNAs expression profiles in *C. elegans* have been well studied in terms of aging [12]. In addition, accumulating evidence indicates that ceRNA network may play important roles in many disease processes [13, 14]. However, the interactions between the mRNA, ncRNA and ceRNA affected by the mitochondria variation in COX1 and the potential roles of the miRNA, lncRNA, circRNA and ceRNA in the pathogenesis of aging are still unclear and have not be characterized yet. Here we used next generation sequencing (NGS) to identify mRNA and ncRNA and evaluate their associations with mitochondria and aging. Differentially expressed mRNA, lncRNA, miRNA, circRNA, and ceRNA were identified between wild strain and cybrid strain along the aging process. Regulation of the transcriptional regulatory networks in the aging process was also investigated in this study.

The molecular and cellular networks associated with aging rely on a large number of proteins that collaborate together to make adaptive and contingent metabolic responses to environmental challenges [15]. So far, several metabolic studies of aging have been reported and a series of age-related metabolites were discovered [16, 17]. Here, we aim to dissect the associations between metabolic regulation and longevity regulated by the natural mitochondrial polymorphism, which will provide translational evidence to promote human health-span at genetic level. We applied a mass spectrometry-based approach (MS) to screen global endogenous metabolites in N2 wild strain and trans-nuclear cybrid strain. Integrative analysis of transcriptome and metabolome revealed that the biological process could be influenced by natural variation in mitochondria and lead to accelerate aging.

Through high-throughput sequencing and metabolomics analysis (Supplementary Figure 1), this study provided an insight into the roles of COX1 variations in aging process through whole-scale integrative transcriptomics and global metabolomics in *C. elegans*, which identified important potential aging targets for future studies in aging intervention and age-related disease prevention.

**Figure 1:**
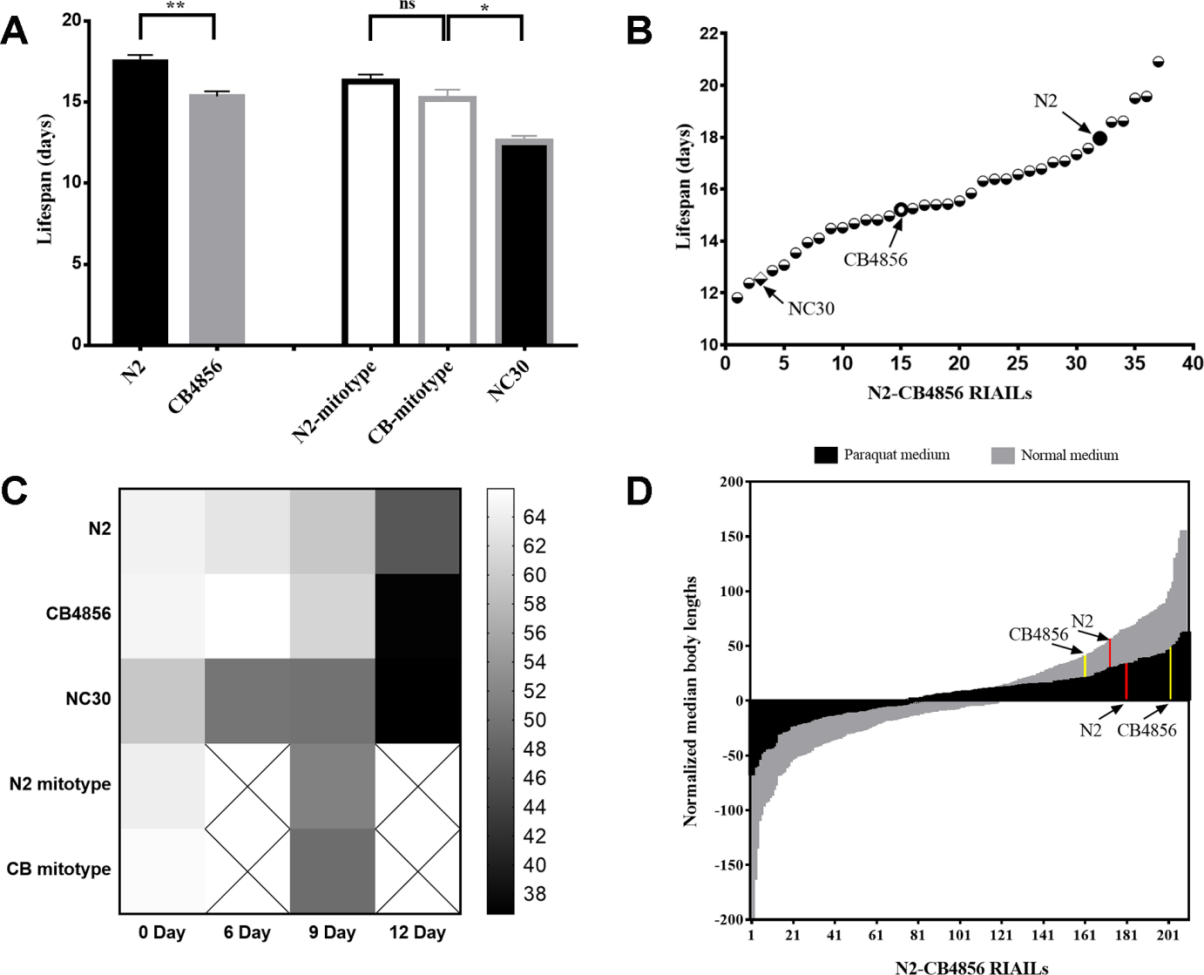
**Both mitochondrial and nuclear genomes correlate with the quantitative traits of development and lifespan of Caenorhabditis elegans.** (**A**) Average lifespans in strains N2, CB4856, N2-mitotype RIAILs, CB4856-mitotype RIAILs and CN30. (**B**) Lifespan of 34 N2-CB4856 recombinant inbred advanced intercross lines (RIAILs). (**C**) Motility of 0, 6, 9 and 12-day-old worms (darker color in the heatmap indicates weaker motility). (**D**) Histogram of normalized body length in control (gray) and in paraquat (black) conditions for 210 RIAILs. Statistical analysis was performed by using two-tailed unequal variant Student’s t-test (* P-value < 0.05, ** P-value < 0.01).

## RESULTS

### Both mitochondrial and nuclear genomes correlate with the quantitative traits of development and lifespan of *C. elegans*

Comparing to wild-type strain N2, a single nonsynonymous SNV occurs in COX1 of the wild-type strain CB4856 via the replacement of alanine with serine (p.A12S) [8]. To determine whether COX1 variation in CB4856 could independently reduce lifespan than N2, the mitochondrial origin and lifespan of the 34 recombinant inbred advanced intercross lines (RIAILs) was quantified. There was no significant difference in the averaged lifespans between CB4856 and N2 mitotypes RIAILs strains. The trans-nuclear cybrid strain CN30 which carried CB4856 mitochondria with N2 nuclear background was then generated, which was verified by 18 SNPs evenly distributed on the six pairs of chromosomes. The lifespan of CN30 was significantly shorter than those strains carrying CB4856 mitochondria but not N2 nuclear background (Figure 1A). In addition, among the 34 RIAILs, 13 strains had a shorter lifespan than CB4856, 5 strains had a longer lifespan than N2, and 16 strains showed intermediate lifespans (Figure 1B). Excessive shorter lifespan of CN30 compared to wild-type strains suggests that there may be some genetic incompatibility between these RIAILs.

Motility is an important physiological and functional indicator of health-span [18, 19]. In this study, motility of 0, 6, 9 and 12-day-old *C. elegans* was quantified according to liquid media spontaneity in wave-like channels. Decline in motility from day 6 was significantly accelerated for CN30 strain when compared with the other two wild-type isolates. The RIAILs that have partial mit-n mismatches also showed faster rates of motility reduction than wild-type isolates (Figure 1C). Previous studies have shown that growth rate (as measured by the length of offspring or optical density) varies substantially in 210 RIAILs with defined nuclear and mitochondrial genotypes [20]. Using these data, we identified 160 RIAILs with relatively lower growth rate compared to that of CB4856, 35 RIAILs with relatively higher growth rate compared to N2, and 12 RIAILs with intermediate growth rate (Figure 1D). Moreover, RIAILs growing in paraquat conditions showed the same trend as described above. These results confirmed that the mit-n mismatches could interfere the development and aging of *C. elegans*.

### Variation in COX1 decrease mitochondrial mass with age

**Figure 2:**
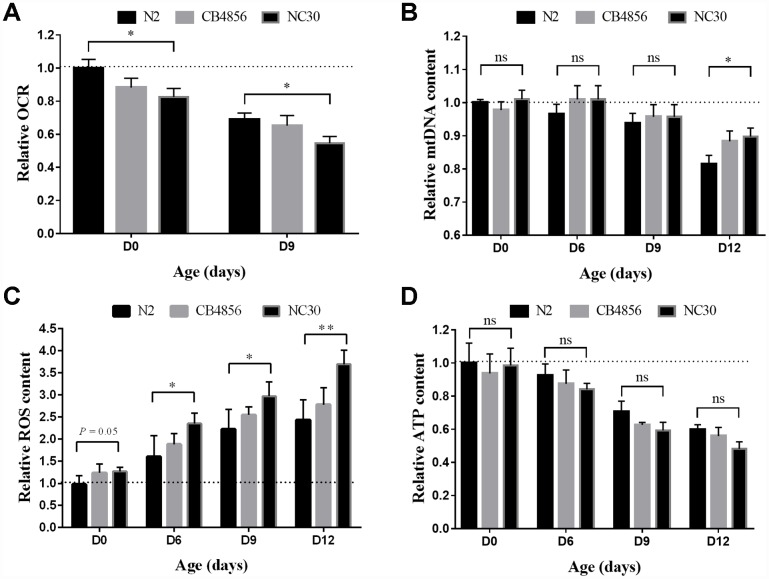
**Variation in COX1 decreases mitochondrial mass with age.** All measured mitochondrial indicators were normalized to the value of day 0 N2 worms. (**A**) Level variation of OCR with age. (**B**) Level variation of mtDNA with age. (**C**) Level variation of ROS with age. (**D**) ATP level variation with age. Data shown as the means ± SEM. Statistical analysis was performed by using two-tailed unequal variant Student’s t-test (* P-value < 0.05, ** P-value < 0.01).

In this study, we examined how the variation in COX1 decrease mitochondrial mass with age. Oxygen consumption rate (OCR) of mitochondria is dependent on many biological reactions and has emerged as a molecular markers for mitochondrial health [21, 22]. Our results showed that OCR decreases with age in N2, CB4856 and CN30 worms, and OCR of CN30 have a greater reduction than N2 (Figure 2A). The relative mtDNA genome content of the two wild-type isolates and the cybrid CN30 strain were assessed by qPCR in 0, 6, 9 and 12 day old worms [9]. There was no difference of mtDNA content among the three strains in 0, 6, and 9 day old worms. However, in the 12-day old worms, N2 strain had a significant reduction in mtDNA content relative to CN30 (Figure 2B). These results suggested that the variation in COX1 led to a faster decline in OCR, which was not related to mitochondria copy number. Although the function of free radical compounds in aging is still controversial, aging is generally accompanied with an increase in reactive oxygen species (ROS) production [23]. Thus, we measured ROS content per worm and found that ROS content was significantly increased with age, especially in CN30 (Figure 2C). In addition, old CN30 worms have more mitochondrial copy number, lower OCR value, and higher ROS content than N2 worms, which suggested that variation in COX1 could reduce mitochondrial functions and lead to aging.

ATP production levels were also examined to determine the quality of mitochondrial functions. Although ATP level decreases with age, the results did not show significant difference among N2, CB4856 and CN30 worms (Figure 2D). In specificity, mtDNA content of CN30 showed smaller downward trend with age-related to N2 strain. ATP levels of CN30 downward trend is more obvious (60% VS 49%). When combined these results together, we can speculate that an organism needs a certain amount of energy to accomplish its basic physiological activities, as COX1 can reduce mitochondrial function, more mitochondria are needed for energy, then more ROS produced by more dysfunctional mitochondria.

### Variation in COX1 linking with nuclear genome SNPs and up-regulate or down-regulate mRNA, lncRNA, miRNA and circRNA profiles

The CN30 and CB4856 worms have the same mitochondria genotype with similar molecular functions but different lifespans, indicating that impaired mitochondrial function caused by COX1 variation alone cannot accelerate the aging process. Thus, nuclear genes associated with aging might be a function of mitochondrial polymorphisms in *C. elegans*. Our previous studies indicated that abundant loci and genes in the nuclear genome might be associated with mitochondria variations and the quantitative traits of lifespan in *C. elegans* [9]. By further comparing the transcriptomes of CN30 and N2 worms with the same nuclear background but different mitochondrial genotypes, we can study how mitochondria up-regulate or down-regulate nuclear genes and then influence the aging process.

Here, we detected which loci and genes were linked with mitochondrial variations by using the high-throughput sequencing data and the published SNPs chip data [10]. Single nucleotide polymorphisms in nuclear genome that might be linked with mitochondrial polymorphisms were searched for by using case/control association option of the PLINK software [30], which could estimate whether nuclear loci have epistasis effects with mitochondrial polymorphisms. In the 93 strains with npr-1 CB4856 genotype, 92 out of 1454 SNPs were found to have linkages with mitochondrial polymorphisms (P < 0.01, q-value <= 10%, Supplementary Table 1). The genotype of most SNPs associated with mitochondria (CB4856 or N2 genotype) matched with the mitochondria genotype (CB4856 or N2) (matched vs unmatched: 62 vs 30, *P* < 0.05, Supplementary Table 1). The results suggested that there exists a correlation between mitochondrial genome and nuclear genome.

**Figure 3:**
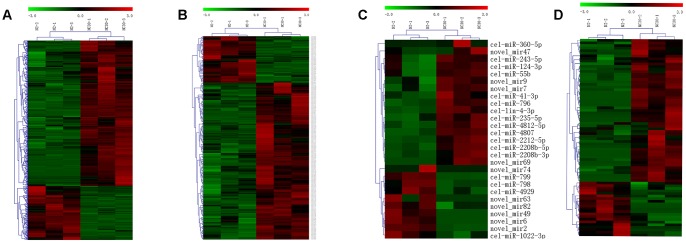
**Influences of variation in COX1 on mRNA, lncRNA, circRNA and miRNA expression profiles.** Heatmaps of the differentially expressed (**A**) mRNAs, (**B**) lncRNAs, (**C**) circRNAs, and (**D**) miRNAs in between CN30 and N2 worms. The data are depicted as matrices in which each row represents one mRNA, lncRNA, miRNA, or circRNA and each column represents one of the worm samples. Relative mRNA, circRNA, miRNA, or lncRNA expression is depicted according to the color scale shown at the top. Red and green colors represent relatively high and low expression values. The magnitude of deviation from the median is represented by color saturation.

The expression profiles of mRNAs and lncRNAs were generated by Illumina X ten in wild strain N2 and the trans-nuclear cybrid strain CN30. The expression profiles of miRNAs were measured by BGISEQ-500. RNAs with a threshold value of fold-change >2 and q-value ≤ 0.001 were identified as significantly differential expression genes. The expression levels of 801 mRNAs, 245 lncRNAs, and 26 miRNAs were significantly altered in CN30 compared to N2 controls (Figure 3A–3C), among which 584 mRNAs, 53 lncRNAs, and 16 miRNAs were up-regulated, while 217 mRNAs, 192 lncRNAs, and 10 miRNAs were down-regulated. CircRNA which is a large class of noncoding RNA and plays important roles in gene expression regulation were also predicted, 76 circRNAs were significantly differentially expressed in between CN30 and N2 worms (Figure 3D). The differentially expressed mRNAs, lncRNAs, circRNA and miRNAs in the CN30 worms are all listed in Supplementary Table 2.

### Age-dependent changes in gene expression

**Figure 4:**
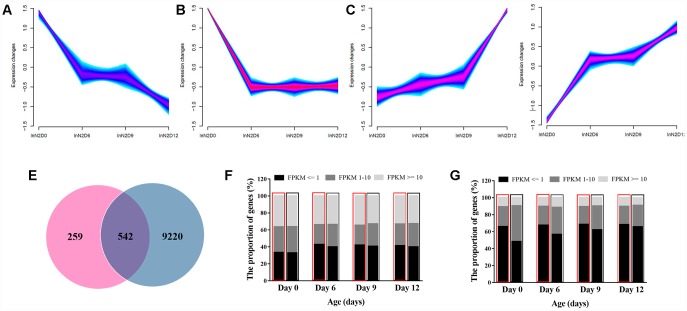
**Age-dependent changes in gene expression levels.** (**A**–**D**) Clusters of genes associated with age. Genes with the same expression pattern were grouped into a cluster. Yellow and green colors correspond to low membership values, while red and purple colors correspond to high membership values. (**E**) Venn diagram of the differentially expressed genes. Each circle represents a set of genes. The left circles represent the genes associated with mitochondria variation. The right circles represent the genes associated with aging. The region superimposed by circles represent the genes both associated with mitochondria variation and aging. (**F**) Gene expression features at the whole genome level. High expression, intermediate expression, and low expression are represented by FPKM>=10, FPKM=1~10 and FPKM<=1, respectively. (**G**) Gene expression features of 920 genes affected by mitochondria polymorphism.

Among the 21473 genes sequenced by Illumina X ten platform, we identified 9762 genes with significantly differential expressions between young and elder worms. We then divided these genes into 4 clusters according similar time-dependent changing trend (Figure 4A–4D). All the genes that regulate aging must change with aging in terms of expression profiles. We then examined whether genes affected by the variation of mitochondria could have a similar correlation with aging. Most of the genes that were associated with mitochondria (Figure 3A) showed age related expression feature (542/801 *vs*. 9762/21473, *P-*value = 3.58E-35, two-tailed Fisher exact test) (Figure 4E). These results suggest a role of the natural variation of mitochondria in aging via regulation of multiple nuclear genes that might link with aging. We also assessed the influence of age at whole genome expression level between N2 and CN30. In developing worms, by analyzing the gene expression level of 21473 genes, more genes showed a high expression level than adult and elder worms (Figure 4F). For 801 genes that were significant up-regulated or down-regulated by mitochondria, the average expression level was significantly lower than the whole genome gene expression level. For CN30 worms, by analyzing the gene expression level of 801 genes, the number of higher expression genes were more than N2 worms (646 vs 274) (Figure 4G). These results suggested that mitochondrial polymorphism lowered basal expression levels of relevant genes and participated in the regulation of aging.

### Interactive network of mRNA, lncRNA, miRNA and circRNA, and ceRNAs

**Figure 5:**
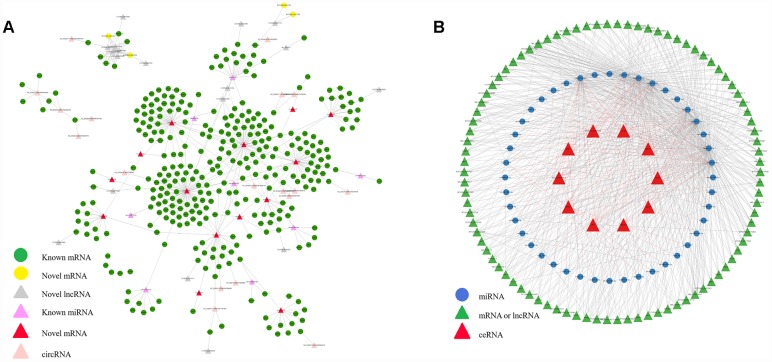
**Interactive network of mRNA, lncRNA, miRNA and circRNA, and ceRNAs.** (**A**) The regulatory network was constructed through visualizing the relationships between differentially expressed circRNA, lncRNA, miRNA and mRNA and their target genes. (**B**) The interaction network among the top 10 ceRNAs network.

To better understand the functions of non-coding RNAs and the corresponding genetic regulatory networks affected by COX1, the mRNA-lncRNA-miRNA-circRNA network was visualized by software CytoScape [24]. The possible miRNA targets (Supplementary Table 3) and the lncRNA targets (Supplementary Table 4) were predict. Relationships between circRNA and lncRNA were also determined by the position of circRNA and lncRNA on chromosomes. Finally, according to the interactions among differently expressed mRNAs, lncRNAs, miRNAs and circRNAs, complex regulatory networks were generated (Figure 5A).

Competing endogenous RNA (ceRNA) can compete with other RNA transcripts for the same microRNA. Hence achievement of mutual communication and regulation. Thus, we predicted the candidate ceRNAs and the top 10 candidate ceRNAs which have the most correlation with other RNAs were selected (Supplementary Table 5). Then, the interaction network between the top 10 ceRNAs, shared miRNAs, and target RNAs was constructed (Figure 5B). Since most of the genes associated with mitochondria showed age related expression feature, studying on the composition of the gene regulatory network is also of great value for aging research.

### Variation in COX1 affects metabolic activity

**Figure 6:**
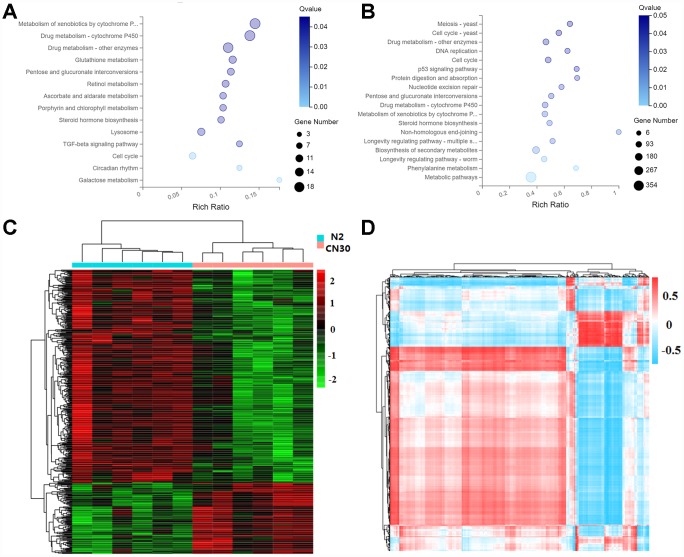
**Variation in COX1 affects metabolic activity.** (**A**) The significantly influenced KEGG pathways by mitochondria variation. X-axis: enrichment radio. Larger value represents greater degree of enrichment. Rich ratio indicates the number of differentially expressed genes located under the path term divided by the number of all annotated genes under the path term. Y-axis: pathway. Color: P-value. Circle size: number of genes. (**B**) Significantly influenced KEGG pathway by age. (**C**) Heat map of differentially expressed metabolites. The data were depicted as matrices in which each row represents one metabolite and each column represents one of the worm samples. Relative metabolite expression is depicted according to the color scale shown at the right side. Red and green colors represent high and low relative expression, respectively. The magnitude of deviation from the median is represented by color saturation. (**D**) Interactions between genes and metabolites both affected by the mitochondria variation. Pearson correlation coefficients that represents the association between genes and metabolites were depicted according to the color scale shown at the right side. Red and blue colors represent positive and negative correlations. The magnitude of deviation from the median is represented by color saturation.

KEGG pathway analysis showed that 14 pathways were significantly altered by genes linked with mitochondria variation (q-value < 0.05), among which 9 pathways were related with metabolic activity (Figure 6A). For age-related genes, 18 pathways were significantly altered by genes linked with age (q-value < 0.05), 5 pathways of which were related to metabolic activity (Figure 6B). These results suggested that the variation in mitochondria regulates aging process mainly by influencing metabolic activity.

We further examined the metabolic changes in the elder worms by using mass spectrometer Xevo, G2-XS QTOF (Waters, UK) for mass spectrometry data collection. Identification of metabolites was based on database KEGG. The variable important for the projection (VIP) values (VIP≥1) of the first two principal components of the multivariable PLS-DA model was used to screen the differential metabolites by combining fold-change (≥1.2 or ≤0.8333) and q-value (<0.05). Analysis of 12 samples (9-day old) yielded 7133 total ions by positive ion mode and relative standard deviation (RSD) <=30%, among which 474 metabolites were differentially regulated between N2 and CN30 (Figure 6C). The metabolic pathways of differential metabolites were further annotated based on the KEGG database (Supplementary Table 6).

In order to integrate the data of differentially expressed genes and differentially present metabolites, we thoroughly analyzed these genes and metabolites involved in the regulation process, which formed a high-confident gene expression regulation network. Cor function in R language was used to calculate the Pearson correlation coefficient between 474 metabolites and 135 genes that were linked with age and were differentially expressed in between CN30 and N2 at both young and elder stages. According to the correlation coefficient, Pheatmap and RColorBrewer packages were used in order to generate the correlation clustering heatmap (Figure 6D) (Supplementary Table 7). These correlation analyses can provide important guidelines for future studies on aging regulation.

## DISCUSSION

Although mitochondrion only harbors a small set of genes compared with the organism's genome, it provides the necessary energy for cells and plays important roles in signal transduction, cell growth, and apoptosis, *etc*. Harman first proposed the "mitochondrial aging theory" in 1956, arguing that mitochondria are the core component of aging that is accompanied by an increase in mitochondrial DNA mutations [23]. Our previous studies have shown that the degree of mismatch between mitochondrial and nuclear genomes may be one of the important factors influencing the differences in lifespans among worm populations [9]. In this study, we showed that most individuals in the RIAILs hybrid population have lower lifespan and development level than their parents. We also confirmed the hypothesis proposed by Dingley *et al.* that mtDNA-nuclear genome mismatch may lead to reduced lifespan of *C. elegans* by using the trans-nuclear cybrid strain which have almost the same nuclear background with the N2 strain [8]. We further studied the various mitochondrial mass in different life stages of worms and explored how mitochondria variation regulated the nuclear genome and influenced the aging process.

The nuclear transfer strain CN30 was constructed by first crossing N2 males with CB4856 hermaphrodite, then by repeatedly crossing the offspring with N2 males up to 20 generations, and finally by inbreeding up to 30 generations to generate the final CN30 strain. The strains were verified by 20 representative SNPs that were evenly distributed on the six pairs of chromosomes and the mitochondrial genome. Since the N2 and CN30 worms have almost the same nuclear background and they were experimented with the standardized and uniform environment, the trans-nuclear cybrid strain CN30 is a good model to study mitochondrial function and its interaction with nuclear genome. In this study, we confirmed the hypothesis proposed by Dingley *et al.* that mtDNA-nuclear genome mismatch may lead to reduced lifespan of *C. elegans* [8]. We further studied the various mitochondrial mass in different life stages of worms and explored how mitochondria variation regulated the nuclear genome and influenced the aging process.

Strain CN30 has a reduced OCR compared to strain N2 in both young and elder worms, which suggests that variation in mitochondria can alter energy metabolism. This is consistent with previous studies [8]. However, there was no difference in ATP content between CN30 and N2 accompany age. Since the process of ATP production is aerobic, there must be more mitochondria with lower OCR in order to produce a certain amount of ATP. Based on this corollary, we further examined the copy number of mitochondrial DNA (mtDNA) in different life stages of worms. We found that mtDNA content has no significant decrease in developing worms. Meanwhile, N2 had a significant reduction in mtDNA content relative to CN30 for the elder worms. The cybrid strain with mit-n mismatch also posted significantly increased CIV activity [8]. By combining these results, we proposed that impaired mitochondrial function need mobilizes the maximum potential of producing ATP and increases quantity to supply the necessary energy for basic life activities. Lifespan is a complex biological trait that is bound to be regulated by a complex network involving many genes. We postulated that mitochondria should be able to not only provide energy for life activities but also supply energy for the gene regulatory networks. Thus, it is valuable to study the mechanism of mitochondrial regulation of aging by exploring the age-related gene regulatory networks affected by mitochondria polymorphisms.

Previous studies suggested that six quantitative trait locus (QTLs) in the worms were associated with lifespan [25]. In all the six QTLs, N2 genotypes have increased lifespan while CB4856 genotypes have decreased lifespan. Strain CNC30 with N2 nuclear background and CB4856 mitochondria have lower lifespan than strain CB4856, which suggests that mitochondrial genotypes also up-regulate or down-regulate nuclear gene expression and in turn decreases worm lifespan. Here we assumed that aging-related genes should be changed along with aging in terms of expression levels. Therefore, we studied the whole-transcriptome changes in gene expression affected by the natural variation of mitochondria and age of *C. elegans*. We identified a group of genes affected by mitochondria polymorphism, most of which showed changed expression levels with age. Since mitochondria consisting of about 1000 proteins and most of them are encoded by nuclear genes, previous studies mainly focused on how nuclear genes affect the aging process [4, 9, 26]. Our results suggested that many genes can be actually affected by mitochondria directly and were involved in aging regulation.

Cumulative evidence indicates that ceRNA and circRNAs can function as endogenous sponges to influence mRNA, lncRNA or miRNA activity [27, 28]. We found many ncRNAs were significant up-regulated or down-regulated by the mitochondria polymorphism in *C. elegans*, which include novel miRNAs and lncRNAs and predicted circRNAs and ceRNAs. The miRNAs-lncRNAs-mRNA-circRNAs gene regulatory network and the ceRNA network affected by mitochondria was constructed. In addition, due to vastly changed environmental stresses, organisms might make corresponding adjustments at molecular level [15] (especially mitochondria variation). Thus, facing the genetic pressure (especially mitochondria variation), gene regulation network that regulates complex traits would also be influenced. The application of predicted circRNAs and ceRNAs and the gene regulatory network analysis may provide a novel view of the interactions between mRNAs and ncRNAs. Our results provided the first assessment of gene regulation networks influenced by mit-n and identified certain known and novel mRNA, lncRNA, miRNA, circRNAs and ceRNAs that were significant up-regulated or down-regulated by mitochondria and involved in aging.

To better understand the biological functions and potential mechanisms of the genes affected by mitochondria and age, we also performed KEGG pathway analysis. The significantly relevant KEGG pathways were metabolic pathways. Combining with the fact that metabolic level could change significantly with age [16], these results indicated that mitochondria variation could change not only the metabolism of the mitochondria but also the metabolism of the whole organism, generating impacts on the aging process. We also examined elder *C. elegans* metabolism and found 474 metabolites changing between N2 and CN30. Most of these metabolites belong to the metabolic pathway. Multi-omics integration analysis between metabolomics and transcriptomics was then performed. Our study established the relationship between the natural mitochondria variation and aging from the perspectives of nuclear genome regulatory network and metabolomics.

## CONCLUSIONS

This study provides important genetic evidence for the hypothesis that mitochondria regulate aging through its functions and effects on nuclear gene regulatory networks. To further verify this hypothesis, the roles of genes and metabolites in the gene regulation network should be investigated and clarified in the aging process. Since promotion of ATP synthesis can help maintain healthy life activities [29], analysis of transcriptome expression after the improved mitochondrial function is also needed for further study.

## MATERIALS AND METHODS

### Strains and media

*C. elegans* RIAILs were gifts from Professor Leonid Kruglyak (Princeton University, Princeton). *C. elegans* were fed with *E. coli* OP50 at 20 °C on normal growth medium (NGM).

### Association mapping

SNPs associated with mitochondrial genotype were analyzed by using software package PLINK (v1.07) with the quantitative trait association option [30]. SNPs in stronger linkage disequilibrium with other SNPs were not removed as all these SNPs can be used to discern the genomic extent of intervals associated with traits [10].

### Lifespan assays

Worm lifespan assays were performed by following the manufacturing instructions and are briefly described as follows. First, 100 larva stage 4 worms were transferred to NGM containing *E. coli* OP50, and three culture dishes were set for each line. Then, worm survival condition was scored every day by observing the pharyngeal movement and the touch-provoked movement using platinum wire. Worms were considered to be dead if there was no pharyngeal movement and no touch-provoked movement. Each experiment was duplicated three times.

### Motility assays

Synchronized worms were separately transferred to food-free 3 ml NGM medium at 20 °C for three minutes. Then, CCD camera was used to record the motility of worms in M9 buffer continuously and the number of head sway from left to right in 30 seconds was also recorded. All the strains of worms used in the study were examined at the same stages and each experiment was repeated three times.

### Assay for the origin of nuclear and mitochondria

Mitochondrial genomes from the two wild worms N2 and CB5856 were compared, and two SNPs with different loci were randomly selected [9]. Then, the mitochondria DNA was extracted and amplified with two pairs of primers (SNP1: AGAATGATTTACGTTACCA/TTATT TTTTTGATTTT, A = N2, T = HW; SNP2; AGAATGA TTTACGTTACCA/TTATTTTTTTGATTTT, A = N2, T = HW). DNA sequences of the nuclear genomes were compared, and 18 SNPs that evenly distributed on the six pairs of chromosomes were selected. Nuclear DNA was extracted and amplified with 18 pairs of primers.

### Measurements of relative mtDNA content per cell.

Real-time PCR was used to determine the relative copy number of mtDNA content per cell [8]. Since nuclear genomic DNA is relatively stable at different stages of life, it was used as a housekeeping gene [31]. The method is briefly described as follows. A single cleaned worm was picked up into a PCR tube containing 10 μ L 100 μ g/ml proteinase K. Then, the PCR tube was tightly sealed, and it was heated at 56 °C for 15 min and 95 °C for 10 min. Then, 10 μ L SYBR Green Supermix (BIO-RAD iQ TM SYBR Green Supermix, cat. No. 170-8882AP) were added to the PCR tube and amplified with primers specific to either nuclear DNA (F, TGGAACTCTGGAGTCACACC, R, C ATCCTCC TTCATTGAACG G) or mtDNA (F, GTTTATGCTGC TGTAGCGTG, R, CTGTTAA AGCAAGTGGACG AG) [31]. The 10 synchronized 0-day, 6-day, 9-day and 12-day old adult worms were examined for each strain and the mean values were used.

### Assays for ATP levels ATP

Levels assays were performed by the manufacturing instructions of ATP bioluminescent assay kit (Sigma, product number FL-AA) [9]. ATP concentration in the synchronized 0-day, 6-day, 9-day and 12-day old adult worms was examined. The ATP content were normalized by the number of worms and each experiment was repeated 3 times.

### Assays for ROS levels

The worms stained with DCFDA solution at a final concentration of 50 μM DCFDA for 30 min at 20°C [32]. Twenty synchronized 0 days, 6 days, 9 days and 12 days old worms were picked up onto microscope slides which coated with 3% agarose and anaesthetized with 2% sodium azide. Worms were photographed by fluorescence microscope (100× magnifications and 200 s exposure time). The fluorescence intensity of all images was calculated by software Image-Pro Plus.

### Assays for the rate of oxygen consumption (OCR) levels

OCR levels for whole *C. elegans* in the synchronized 0-day, 6-day, 9-day and 12-day old adult worm were examined using an analyzer Seahorse XF24 [33]. All the Worms were washed 4 times in M9 buffer to segregate clean bacteria-free adult worms from bacterial debris. Worms were loaded about 30 worms to each well in M9 buffer. Three OCR readings were taken after 20 min equilibration. Data was normalized by the number of worms per well and each experiment was repeated three times.

### Total RNA extraction and non-coding RNA library construction

Total RNA was extracted from the thousands of synchronized worms using Trizol (Invitrogen, Carlsbad, USA) according to the manufacturing instructions. RNA was qualified and quantified using a Agilent 2100 and NanoDrop bioanalyzer (Thermo Fisher Scientific, USA). Qualified lncRNA libraries were sequenced via pair-end sequencing on the Hiseq xten platform [34]. Qualified small RNA libraries via pair-end sequencing on the BGISEQ-500 platform (BGI-Shenzhen, China) [35].

### lncRNA-seq data analysis

Raw reads with rRNA, low quality, joint contamination, and high content of unknown base Ns were filtered out (Supplementary Table 8). Then, clean reads were matched to the reference genome using HISAT [36] and assembled using StringTie [37]. After obtaining the new transcript, the mRNA and lncRNA were distinguished by using three software CPC [38], txCdsPredict, CNCI [39] and the database Pfam [40]. Scores should exceed the threshold (CPC_threshold = 0, CNCI_threshold = 0, txCdsPredict_threshold = 500). If transcripts can be compared to Pfam database, they were conserved to be mRNA, otherwise they were conserved to be lncRNA. At least three of the four criteria were consistent, and then it was confirmed that the transcript was an lncRNA or mRNA. NT, NR, KOG, KEGG, and SwissProt were used for annotation by software Diamond [41]. GO annotation was performed by using NR annotation results and the Blast2GO software [42]. Clean reads were compared to the reference sequence by using the Bowtie 2 software [43]. Then software of RSEM that a model generated by reads as used to calculate the genes expressions [44]. Transcripts and clean reads were allocated to different transcription by using maximum likelihood (ML) method. The chain-specific model used to distinguish the reads from the positive and negative strands could facilitate a more accurate quantitative analysis of gene expression. The expression levels of genes were standardized by FPKM. Differential genes between groups were analyzed via software DEGseq [45]. Genes with change fold >= 2 and q-value <= 0.001 were identified as with significantly different expression levels between groups in this study.

### Small RNA-seq data analysis

The impurities of raw data are 5' primer contaminants, no insert tags, oversized insertion, low quality tags, poly A tags and small tags, among which no insert tags and 5' primer contaminants are defined as adaptor contaminants, oversized insertion manifests as missing 3' primer. Generally, adaptor contaminant is related to the sample itself and the density proportion between the adaptor and the sample. The higher the proportion is, the worse the contaminant is. Low quality tags which have more than four bases whose quality is less than ten and which have more than six bases whose quality is less than thirteen will be filtered to ensure that the tags to analyze are reliable. We will get rid of the above contaminant tags and low-quality tags from the FASTQ file and get the final clean tags. After filtering, clean tags were mapped to sRNA database. The characteristic hairpin structure of miRNA precursor can be used to predict novel miRNA. We use miRDeep 2 [46] to predict novel miRNA by exploring the secondary structure, the Dicer cleavage site and the minimum free energy of the unannotated sRNA tags which could be mapped to the genome. The small RNA expression level is calculated by using TPM [47].

### The time series analysis of gene expression

The time series analysis of gene expression was carried by the software Mfuzz [48]. At the same time, genes with silimar expression may take part in the same biological function and help to discover regulatory genes. The membership value of Mfuzz which is used to evaluate whether the gene conforms to the trend of the gene cluster is the value between 0 and1. The membership value of the gene closer to 1, the more the trend of the gene is consistent with the trend of the gene cluster.

### KEGG pathway analysis

KEGG pathway were used to divide the functional modules of the genes. If the candidate gene is significantly enriched in a functional module, the proportion of the candidate genes in the functional module is significantly higher than the overall genes. Each functional module has a p-value, and the smaller the p-value, the richer the candidate genes are in this functional module. Then q-value correction was performed on p-value, and the function of q-value <= 0.05 was set as significant enrichment.

### lncRNA target prediction

The function of lncRNA is mainly achieved by acting on the target gene by cis or trans. lncRNA was determined as cis within 10k upstream of mRNA or 20k downstream of mRNA. Beyond this range, RNAplex was used to analyze the binding energy of lncRNA and mRNA [49]. If the binding energy was less than -30, it was considered to be trans. The two correlation coefficients of lncRNA and mRNA, spearman and Pearson, were calculated. Spearman_cor >= 0.6 and Pearson_cor >= 0.6 mean that there is a correlation between lncRNA and mRNA.

### miRNA target prediction

In order to find possible miRNA targets, multiple software was used. Taking the intersection targets with appropriate filter conditions such as MFE, score for further analysis. Generally, we use RNAhybrid [50], miRanda [51] and TargetScan to predict miRNA target.

### ceRNAs prediction

The significant miRNAs shared between candidate ceRNA and mRNA were calculated, and the significant ceRNAs were predicted by combining the ratio of the shared miRNA by ceRNA and miRNA, the ratio of the shared microRNA response element (MRE) by miRNA and ceRNA, the density of shared MRE by ceRNA and mRNA, and the evenness of the shared MRE by ceRNA and mRNA.

### CircRNA prediction and annotation

This study used CIRI [52] and find_circ [53] to predict circRNA, and integrated the results based on the start and end positions of the circRNA (both the start and end positions are within 10 bases before and after the circRNAs are merged into one class). CIRI is a software developed based on the BWA-MEM genome alignment algorithm, which detects circRNA by scanning the generated SAM (Sequence Alignment/Map) file. The two parts before and after the same read need to be cross-aligned to the genome in order to prove that they are junction reads at both ends of the circRNA. Therefore, during the first pass, CIRI will filter the junction reads with paired chiastic clipping (PCC) signals and then limit the range of paired-end mapping (PEM). The GT-AG shear signal is further filtered for the junction reads. In the second scan of the SAM file, CIRI will detect new junction reads and eliminate false positive circRNAs caused by mismatches to homologous genes or repeats, thereby improving the sensitivity and accuracy of the software [52]. Please refer to Figure 3 for the specific process.

### Gene regulatory network construction

The circRNA-lncRNA-miRNA-mRNA network was constructed and visually displayed by using CytoScape software [24]. The top 10 candidate ceRNAs which have the most correlation with other RNAs were selected for visualization in the interaction network between ceRNAs, shared miRNAs, and target RNAs. The edges of the network reflect the interactions among different expressions of circRNA, lncRNA, miRNA, mRNA, ceRNAand their target genes.

### Metabolomics analysis

Whole-scale metabolites were detected via mass spectrometer Xevo, G2-XS QTOF (Waters, UK) and identified based on the database KEGG. Software Progenesis QI (version 2.2) (Waters, UK) and R package metaX were used for statistical analysis of mass spectrometry data [54]. Variable Importance in the Projection (VIP) of the first two principal components in the multivariable PLS-DA model was used to screen differential metabolites by combining fold-change and q-value values. The VIP-score is a quantitative measure that indicates the strength and explanatory ability of each metabolite on the classification discrimination of samples in each group. Screening conditions are VIP≥1, fold-change ≥1.2 or ≤0.8333 and q-value<0.05. Metabolites satisfy the three conditions were identified as differential metabolites.

### Association analysis between metabolites and genes

The cor function in R language was used to calculate the Pearson correlation coefficient between the metabolites and the genes that are differentially expressed between CN30 and N2. According to the above correlation coefficient, Pheatmap and RColorBrewer packets are used for cluster analysis, the result of which was used for the correlation matrix heatmap visualization.

### The parameter description of the bioinformatics software used in this study

The parameters of the software were listed in Supplementary Table 9.

### Ethical approval

This article does not contain either human nor animal experiments.

## Supplementary Material

Supplementary Figure 1

Supplementary Table 1

Supplementary Table 2

Supplementary Table 3

Supplementary Table 4

Supplementary Table 5

Supplementary Table 6

Supplementary Table 7

Supplementary Table 8

Supplementary Table 9
