# Supplementary figures and images for: The whole transcriptome regulation as a function of mitochondrial polymorphisms and aging in Caenorhabditis elegans
Source: Aging (Albany NY). 2020 Feb 4;12(3):2453–70. doi: 10.18632/aging.102754 (PMC7041728; doi:10.18632/aging.102754)

SUPPLEMENTARY FIGURE

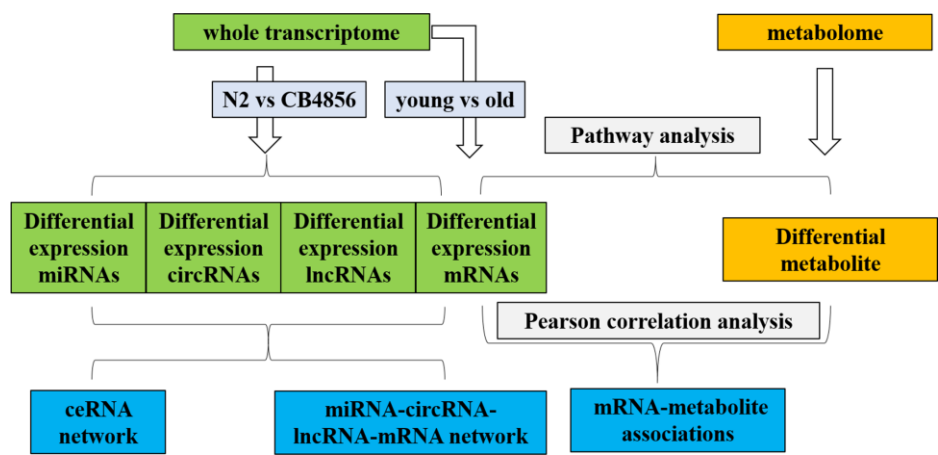

Supplementary Figure 1. The experimental analysis process.

Supplement: Supplementary Figure 1 [file aging-12-102754-s007..pdf]
