# Supplementary material for: The whole transcriptome regulation as a function of mitochondrial polymorphisms and aging in Caenorhabditis elegans
Source: Aging (Albany NY). 2020 Feb 4;12(3):2453–70. doi: 10.18632/aging.102754 (PMC7041728; doi:10.18632/aging.102754)
Supplement: Supplementary Table 9 [file aging-12-102754-s002..docx]

HISAT2:

Version: v2.0.4

Parameter: --phred64 --sensitive --no-discordant --no-mixed -I 1 -X 1000

Website: <http://www.ccb.jhu.edu/software/hisat>

StringTie:

Version: v1.0.4

Parameter: -f 0.3 -j 3 -c 5 -g 100 -s 10000 -p 8

Website: <http://ccb.jhu.edu/software/stringtie>

Cufflinks:

Version: v2.2.1

Parameter: -p 12

Website: <http://cole-trapnell-lab.github.io/cufflinks>

pfam_scan.pl:

Version: 无

Parameters: defaultParameter

Website: <http://pfam.xfam.org/>

CPC :

Version: v0.9-r2

Parameters: defaultParameter

Website: http:// CPC .cbi.pku.edu.cn

CNCI:

Version: 无

Parameters: defaultParameter

Website: <https://github.com/www-bioinfo-org/CNCI>

txCdsPredict:

Version: 无

Parameters: defaultParameter

Website: <http://hgdownload.soe.ucsc.edu/admin/jksrc.zip>

Bowtie2:

Version: v2.2.5

Parameter: -q --phred64 --sensitive --dpad 0 --gbar 99999999 --mp 1,1 --np 1 --score-min L,0,-0.1 -I 1 -X 1000 --

no-mixed --no-discordant -p 1 -k 200

Website: <http://bowtie-bio.sourceforge.net/bowtie2/index.shtml>

RSEM:

Version: v1.2.12

Parameter: --forward-prob 0

Website: <http://deweylab.biostat.wisc.edu/rsem>

pheatmap:

Version: 1.0.8

Parameters: defaultParameter

Website: <https://cran.r-project.org/web/packages/pheatmap/index.html>

Mfuzz:

Version: v2.34.0

Parameters: defaultParameter

Website: <http://mfuzz.sysbiolab.eu>

InterProScan5:

Version: interproscan-5.11-51.0

Parameters: defaultParameter

Website: <https://code.google.com/p/interproscan/wiki/Introduction>

Diamond:

Version: v0.8.31

Parameters: defaultParameter

Website: <https://github.com/bbuchfink/diamond>

Blast2GO:

Version: Blast2GO Pipeline Version 2.5.0

Parameters: defaultParameter

Website: <https://www.blast2go.com>

CIRI:

Version: v2.0.5

Parameters: default

Website: <https://sourceforge.net/projects/ciri/>

miRanda:

miRanda: -en -20 -strict

RNAhybrid: -b 100 -c -f 2,8 -m 100000 -v 3 -u 3 -e -20 -p 1 -s 3utr_human

psRobot: -gl 17 -p 8 -gn 1

TargetFinder: -c 4

TAPIR: --score 5 --mfe_ratio 0.6
